# Introduced beaver improve growth of non‐native trout in Tierra del Fuego, South America

**DOI:** 10.1002/ece3.6636

**Published:** 2020-08-17

**Authors:** Ivan Arismendi, Brooke E. Penaluna, Carlos G. Jara

**Affiliations:** ^1^ Department of Fisheries and Wildlife Oregon State University Corvallis OR USA; ^2^ USDA Forest Service Pacific Northwest Research Station Corvallis OR USA; ^3^ Instituto de Ciencias Marinas y Limnológicas Facultad de Ciencias Universidad Austral de Chile Valdivia Chile

## Abstract

Species introductions threaten ecosystem function worldwide, and interactions among introduced species may amplify their impacts. Effects of multiple invasions are still poorly studied, and often, the mechanisms underlying potential interactions among invaders are unknown. Despite being a remote and well‐conserved area, the southern portion of South America has been greatly impacted by invasions of both the American beaver (*Castor canadensis*) and Brown Trout (*Salmo trutta fario*). Here, we compared growth, condition, diet, and stable isotopes of sulfur δ^34^S, nitrogen δ^15^N, and carbon δ^13^C for stream‐living Brown Trout from streams with (*n* = 6) and without (*n* = 6) beaver in Tierra del Fuego, Chile. We show that beaver may facilitate the success of trout by positively influencing fish growth. Beaver indirectly provide greater food subsidies (i.e., macroinvertebrate abundances) by modifying the local aquatic environment through active dam and lodge building suggesting a one‐way positive interaction. Trout in beaver‐influenced streams occupied a slightly higher trophic level with more depleted sulfur and carbon isotopic ratios suggesting that food web pathways rely on secondary production from autochthonous origin. Trout in beaver‐influenced streams had a wider dietary breadth with diptera and amphipoda as the prey items providing most of the energy, whereas in streams without beaver, trichoptera were the main source of energy for trout. Ultimately, we find that these two species, which have never co‐occurred naturally, bring about the same ecosystem function and the beneficial influences in their native ranges as in invaded systems.

## Graphical abstract

Species introductions threaten ecosystem function worldwide, and interactions among introduced species may amplify their impacts. The southern portion of South America has been greatly impacted by invasions of both the American beaver (*Castor canadensis*) and Brown Trout (*Salmo trutta fario*). We show that beaver may facilitate the success of trout by positively influencing fish growth.

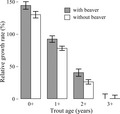

## 1 INTRODUCTION

Species introductions are a major cause of biodiversity loss, and disruption of natural ecosystem processes worldwide (Vitousek, Mooney, Lubchenco, & Melillo, 1997). Currently, biological invasions occur at such high rates in ecosystems across the globe that it is becoming increasingly important to understand the interactions between co‐occurring invaders (Simberloff et al., 2013). Arguably, synergistic interactions are the most important to recognize because they may lead to *invasional meltdowns* where the establishment of one invader facilitates the success of further invasions leading to community‐level responses (Simberloff, 2006; Simberloff & Von Holle, 1999). The ability of some invaders to facilitate other exotic colonizers has been reported through a variety of organisms and pathways, including mutualism, selective herbivory, and facilitation. Mutualism may be a main mechanism for seed dispersal between introduced plants and exotic vertebrates (Richardson, Allsopp, D’Antonio, Milton, & Rejmanek, 2000). Selective herbivory by introduced geese may promote the dispersal of an exotic grass (Best & Arcese, 2009). Predation by an introduced coastal crab on a native bivalve may facilitate the spread of an exotic bivalve (Grosholz, 2005). Aside from these examples, impacts of multiple invaders in natural ecosystems remain poorly explored (Adams, Pearl, & Bury, 2003; O’Dowd, Green, & Lake, 2003). Recently, a meta‐analysis by Jackson (2015) shows that most interactions are either neutral where invasive species do not affect each other, or negative (antagonistic) where the net effect of introduced species is less than the sum of their independent effects. Only a few cases have attempted to identify specific mechanisms underlying interactions between invaders (e.g., Best & Arcese, 2009).

Here, we evaluate ecological interactions between the American beaver (*Castor canadensis*) and Brown Trout (*Salmo trutta fario*) in southern South America. Although these two species have never been in sympatry under natural conditions, they have become dominant invaders co–occurring over the past eight decades on the island of Tierra del Fuego (Arismendi et al., 2019; Lizarralde, 1993; Moorman, Eggleston, Anderson, Mansilla, & Szejner, 2009; Pietrek & González‐Roglich, 2015; Traba & Ríos, 1985). There are, however, coexisting life histories between beaver and salmonids in both of their native ranges. These interactions have shown no overall negative effects on salmonids (Ecke et al., 2017). Indeed, the European Beaver (*C. fiber*) naturally coexists with Brown Trout and Atlantic Salmon (*S. salar*) in Europe. Similarly, the American beaver coexists with many salmonids across North America including Coho Salmon (*Oncorhynchus kisutch*), which has a particularly close relationship with beaver (Leidholt–Bruner et al. 1992). In many cases, there is evidence of beneficial influences from beaver toward salmonids (Leidholt–Bruner et al. 1992; Hägglund & Sjöberg, 1999; Sigourney, Letcher, & Cunjak, 2006).

We hypothesize that invasive American beaver may have an indirect positive influence on growth, condition, and diet of invasive Brown Trout, which should also be reflected in stable isotope ratios. As ecological engineers, beaver cause significant local changes to their environments (Pollock et al., 1995; Wright, Gurney, & Jones, 2004) leading to increased retention of organic matter (Ulloa, Anderson, Ardón, Murcia, & Valenzuela, 2012) and enhanced food subsidies (McDowell & Naiman, 1986; Rolauffs, Hering, & Lohse, 2001). As the top predator in these streams, we expect no differences in δ^15^N values of trout in streams with or without beaver. However, we expect that trout will become increasingly depleted in carbon δ^13^C and sulfur δ^34^S in beaver‐influenced streams due to an increased autochthonous production and bacterial activity in beaver ponds (Carr et al., 2017; McCutchan, Lewis, Kendall, & McGrath, 2003). Beaver dams and their subsequent impoundments have strong implications for macroinvertebrate communities that will likely benefit trout. However, trout with fast growth rates do not always fare well owing to naturally fluctuating environmental conditions including seasonal environments and predation (Biro, Abrahams, Post, & Parkinson, 2006; Carlson, Olsen, & Vøllestad, 2008). Because growth and condition of trout are influenced by the quality and amount of food resources, we characterize the availability and energy content of food. Collectively, our study provides insights about the role of an invader's ecosystem function in facilitating additional invasions, especially, where there are similar histories of coexistence between related species.

## 2 METHODS

### 2.1 Beaver and trout introductions in southern South America

**Figure 1:**
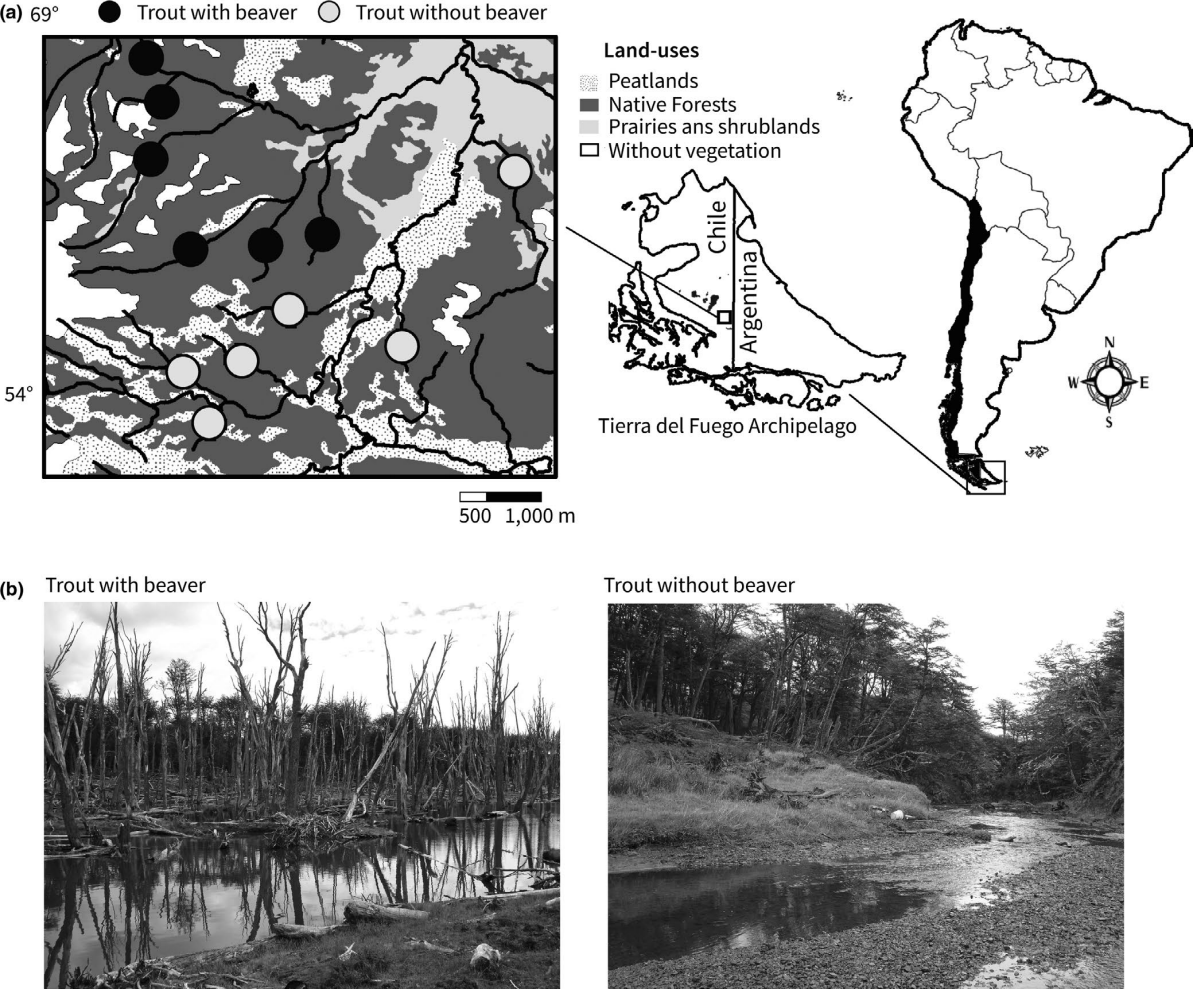
(a) Map of the study area in Tierra del Fuego, South America. (b) Typical stream invaded by Brown Trout and American beaver (left) and stream with Brown Trout, but without beaver (right). Photograph credits: I. Arismendi

In 1946, twenty‐five pairs of American beaver (*Castor canadensis*) were introduced to the southern tip of South America on the island of Tierra del Fuego to develop a felt industry and since, the population has expanded to cover the entire island (Lizarralde, 1993; Pietrek & González‐Roglich, 2015). The Chilean side of Tierra del Fuego alone is estimated to have 41,000 beaver (Skewes et al., 2006). The impacts of beaver are recognized as widespread and visually dramatic across the area transforming 2%–15% of forested areas into impoundments and meadows and altering between 30% and 50% of stream length (Anderson, Martinez‐Pastur, & Lencinas, 2009; Figure 1). On neighboring islands, beaver seem to facilitate the existence of terrestrial invaders (Crego, Jiménez, & Rozzi, 2016) including muskrats (*Ondatra zibethicus*), which in turn sustain American mink populations (*Neovision vison*).

Trout were introduced to southern South America earlier than beaver in the early 1900s by government initiatives to enhance recreational fishing and early practices of aquaculture (Arismendi et al., 2014; Basulto, 2003). Since their introduction, Brown Trout (*Salmo trutta*) have formed naturalized populations and with Rainbow Trout (*Oncorhynchus mykiss*) have become the most abundant fishes in rivers of southern Chile accounting for more than 95% of total biomass (Soto et al., 2006). In the southernmost catchments of South America, Brown Trout are mainly found in streams with very few, if any, Rainbow Trout or native fishes (Arismendi et al., 2019; Soto et al., 2006).

### 2.2 Study area and fish sampling

We conducted field surveys in the Rasmussen River basin located in Karukinka Park in Tierra del Fuego, Chile (Figure 1). This region is composed mainly of well‐conserved habitats offering an ideal environment to understand complex interactions under natural conditions (Smith, 1985). It is part of a trans‐Andean transitional climate with influences of a rainy/oceanic front from the West and the cold tundra from the East (Pisano, 1977). Tierra del Fuego is part of the temperate coastal rivers’ ecoregion (Abell et al., 2008). The mean annual air temperature is 4.5ºC with an annual precipitation range between 600 and 800 mm (Holdgate, 1961). Typical seasonal regimes of stream temperature and discharge are included in Appendix S1. The vegetation is dominated by native deciduous forests of Lenga (*Nothofagus pumilio*) and peatland (*Sphangum* spp.) (Mella, 1994; Pisano, 1977). Biological activity of soils is limited by their acidity (pH 4.0–4.5), low water‐retention capacity, and low nutrient concentrations (Gerding & Thiers, 2002; Romanyà, Fons, Sauras‐Yera, Gutierrez, & Vallejo, 2005). As a result, freshwater ecosystems in Tierra del Fuego are oligotrophic with a very low concentration of nutrients (Perakis & Hedin, 2007). Data available from the nearest operating stream gauging station reveal that high flow conditions occur in winter and spring (snowmelt) and mean daily stream temperature does not exceed 16 ºC during summer (Appendix S1).

The Rasmussen River is dominated by introduced Brown Trout with a rare occurrence of introduced Rainbow Trout and no other introduced or native fishes (Soto et al., 2006; Vila, Fuentes, & Saavedra, 1999). In each stream, we prescreened for the presence/absence of active beaver dams in a 1,000 m transect along the bank by direct observation (complete dam with recent evidence of vegetation cutting by beaver in riparian zone; Figure 1). Streams with any past evidence or incomplete dams were not considered in this study. The distribution of beaver dams across the landscape is uneven (Pietrek & González‐Roglich, 2015) making true replication logistically unfeasible.

After prescreening our study area, we made our best efforts to select relatively comparable study sites (Figure 1; Table 1). Each stream section was classified into one of two categories including Brown Trout in sympatry with American beaver (*n* = 6) or Brown Trout in allopatry with American beaver (*n* = 6). We sampled in the Austral summer (2009–2010) when maximum biological activity occurs in the region (Smith, 1985). Because new beaver dams and lodges are often built in late summer or fall (Lizarralde, 1993), surveyed dams were active for at least a year at the time of sampling. We captured trout from streams using a standard two‐pass backpack electrofishing technique (Bohlin, Hamrin, Heggberget, Rasmussen, & Saltveit, 1989), and to ensure equal fishing effort among streams, we used the same operator and time effort during the study. To account for different‐sized fish, we sampled in all available and accessible mesohabitat units (pool–run–riffle). We recorded total length (TL; cm) and weight (g) of each trout captured.

**Table 1:** Available water quality information from reference freshwaters in Tierra del Fuego and habitat characteristics of our study sites. Numbers represent mean (min, max) values

Parameter	Tierra del Fuego Rivers (annual)	Study sites (summer)	
		Without beaver	With beaver
Stream temperature (°C)^a^	7.0 (0, 16)	9.4 (7.0, 13.8)	11.4 (9.3, 14.1)
Conductivity (µS/cm)^a^, ^c^	117 (35, 720)	93 (77, 102)	106 (10, 176)
Elevation (m.a.s.l.)^a^		206.3 (140, 307)	206.2 (174, 304)
Water velocity (m/s)^c^		0.38 (0.16, 0.64)	0.02 (0.01, 0.03)
Reach—Length (m)^c^		106 (100, 120)	108 (80, 130)
Reach—Width (m)^c^		5 (1.6, 11)	7 (8, 20)
Reach—Depth (m)^c^		0.2 (0.1, 0.8)	1.2 (0.5, 1.5)
Habitat—Eddy (%)^c^		5 (1, 7)	0 (0, 0)
Habitat—Riffle (%)^c^		31 (20, 42)	0 (0,0)
Habitat—Run (%)^c^		25 (20, 40)	2 (1, 8)
Habitat—Pool (%)^c^		38 (30,65)	65 (50, 92)
Habitat—Large wood (%)^c^		1 (0.1, 2)	33 (12, 45)
NO_3‐_ (µg N/L)^b^	0.02		
NH_4+_ (µg N/L)^b^	0.50		
DIN (µg N/L)^b^	0.50		
DON (µg N/L)^b^	19.00		
TDN (µg N/L)^b^	19.50		
DOC (mg C L^−1^)^b^	0.50		

^a^ Dirección General de Aguas, Chile https://snia.mop.gob.cl/BNAConsultas/reportes.

^b^ Perakis and Hedin (2007).

^c^ Present study.

### 2.3 Condition and growth of trout

We estimated the condition of trout using both the index of relative weight (*W*_r_) and the Fulton's condition factor (*K*) as follows:

$$W_{r}=\frac{W_{i}}{W_{s}}\times 100$$

(Wege and Anderson 1978)

$$K=\frac{W_{i}}{L_{i}^{3}}\times 100$$

(Ricker 1975); where *W_i_* is the trout weight at time of capture (g), *W*_s_ is the standard weight for a trout of the same length, and *L_i_* is the trout total length (cm). The *W*_s_ estimation is based on the equation reported for Brown Trout in lotic ecosystems (Milewski and Brown 1994).

The age and relative growth rate (RGR) of trout were estimated by sampling scales from each individual fish and back‐calculating the growth increment from the last winter to the time of capture. We sampled scales of captured trout in sympatry (*n* = 112) and allopatry (*n* = 139). To age trout, we identified annuli with the conventional technique (Chugunova 1959; Davis and Light 1985) of assigning crowding and narrowing of intercirculi distances associated with a lack of growth typical during winter to yearly events. Scales were removed from an area below the posterior margin of the dorsal fin and approximately five scale rows above the lateral line (Pascual et al. 2001). We discarded any resorbed, abnormal, regenerated scales from posterior analysis. To read the scales, we used a microfiche projector, Model II Mini Cat, with a 22 mm lens under 42× magnification. Two experienced scale readers counted and averaged the number of scale annuli from five scales per fish. In case of disagreement, we assigned the age as the mean value between the two readers. We did not find differences on spaced circuli between annuli (Davis and Light 1985), and thus, we did not consider potential anadromy among sampled trout.

We back‐calculated the trout length at the annulus formation according to the Fraser–Lee method (Fraser 1916; Lee 1920) as follows:

$$TL_{i}=c+\left(TL_{c}-c\right)\;\left(S_{i}/S_{c}\right)$$

(Fraser 1916; Lee 1920) where TL*_i_* is the back‐calculated trout length at age *i*, TL_c_ is the trout length at capture, *S_i_* is the mean scale length at annulus, *S*_c_ is the mean scale total length, and *c* is the intercept of the regression between the trout length and mean scale length.

We estimated the relative growth rate (RGR) from the last winter to the time of capture as follows:

$$RGR=\frac{\left(TL_{i+1}-TL_{i}\right)}{TL_{i}}\times 100$$

(Ricker 1975) where TL*_i_* is the back‐calculated total length of trout at the last winter annulus formation and TL*_i_*_+1_ is the total length at time of capture. We used RGR because it is the most recommended calculation of fish growth rate over a short time period (Isely and Grabowski 2007).

### 2.4 Macroinvertebrate densities

In each stream, we deployed a modified Hess sampler 5–8 times for each mesohabitat type (pool–run–riffle) and pooled across type for that stream. Samples were filtered (mesh size of 0.5 µm) and stored in 70% ethanol. In the laboratory, we classified macroinvertebrates to lowest possible taxa. We estimated the density of each macroinvertebrate group (ind./m^2^) for each mesohabitat type by dividing the number of individuals in each macroinvertebrate group by the area sampled for each mesohabitat type. To estimate the density of each macroinvertebrate group from each stream, we simply multiplied the density of each macroinvertebrate group for each mesohabitat type by the proportion of presence for that mesohabitat type in the sampled stream and summed the resulting macroinvertebrate group density values.

### 2.5 Analyses of diets

We subsampled trout stomachs for the dietary analysis in sympatry (*n* = 101) and allopatry (*n* = 186). The stomachs were collected and stored in 70% ethanol. Dietary composition was classified into 19 autochthonous and allochthonous prey categories based on Domínguez and Fernández (2009). Autochthonous prey categories included Diptera, Ephemeroptera, Hirudinea, Plecoptera, Trichoptera, Coleoptera, Hemiptera, Hidracarina, Odonata, Amphipoda (*Hyalella *spp.), Ostracoda, Gastropoda, Chlorophyta, and fishes (trout). Allochthonous prey categories included Coleoptera, Hemiptera, Hymenoptera, Arachnida, and other (vegetation–detritus). We expressed the diet composition using the frequency of occurrence (%*O*), defined as the number of stomachs containing one or more individual prey categories divided by the total number of stomachs analyzed and multiplied by 100; and the percentage by weight (%*W*) defined as the total weight of the prey category divided by the total weight of all prey categories and multiplied by 100 (Hyslop 1980). Trout with a percentage of weight <0.1 (%*W*) were considered to have empty stomachs and were excluded from our final data set.

We estimated the total energetic food consumption of trout by transforming the stomach contents to energetic values. We multiplied the weight of each prey category (g) by its energetic density (cal/g). We converted preserved weights to wet weights using published literature (Donald and Patterson 1977; Leuven et al. 1985) and then converted those corrected weights to energy units with established caloric density values for each prey type (Cummins and Wuycheck 1971). Some taxa did not have caloric values found in the literature, and, consequently, we relied on the nearest taxonomic group possessing a similar morphology (Johnson, Blumenshine, & Coghlan 2006).

### 2.6 Stable isotope analyses

To examine potential differences in trout food sources in sympatry and allopatry with beaver, we analyzed stable isotopes of carbon (C), nitrogen (N), and sulfur (S). We sampled trout of varying sizes (8–25 cm TL) and collected a dorsal portion of muscle. All the tissues were frozen and stored for transportation, dried at 60°C for 48 hr, and ground into a fine powder. All samples were analyzed at the Colorado Plateau Stable Isotope Laboratory, Northern Arizona University. Isotope ratios were expressed in parts per thousand (‰) according to the equation:

$$X=\frac{\left(R_{\mathrm{sample}}-R_{s\tan dard}\right)}{R_{s\tan dard}}\times 10^{3}$$

where *X* is δ^13^C, δ^34^S or δ^15^N and *R* is the corresponding ^13^C:^12^C, ^34^S:^32^S, or ^15^N:^14^N ratio. The standards used were Vienna Pee Dee Belemnite for C, Canyon Diablo Triolite for S, and N_2_ for N. Higher delta values indicated enrichment of the heavier isotope, and lower values indicated depletion. We corrected carbon isotopic ratios due to potential lipid issues following Kiljunen et al. (2006).

### 2.7 Statistical analyses

We analyzed whether growth of trout was a function of the presence of beaver or trout age using a general linear mixed model (GLM) that accounted for streams as a random effect. We fit a model with presence of beaver and trout age as fixed effects. Because the beaver dams create impoundments that should result in a greater availability of food resources (McDowell & Naiman, 1986; Rolauffs et al., 2001), we predicted higher growth of trout from streams in sympatry with beaver. We also separately analyzed whether trout condition (*W*r or *K*) was a function of the presence of beaver and trout age with a GLM that accounted for streams as a random effect. We fit a model with presence of beaver and trout age as fixed effects. We similarly predicted higher condition (*W*r or *K*) for trout at sites in sympatry with beaver. For growth, we analyzed trout ages 0^+^ to 3^+^, but for condition (*W*r or *K*) we excluded age 0^+^ due to the higher uncertainty of weight measurements in smaller trout. In addition, we analyzed whether the energetic food consumption was a function of the presence of beaver or trout age using a GLM that accounted for streams as a random effect. We fit a model with presence of beaver and trout age as fixed effects. We predicted higher energetic food consumption of trout from streams in sympatry than in allopatry with beaver because beaver impoundments likely represent habitats with enhanced food resources. We performed these statistical analyses using PROC GLM in SAS software 9.3 (SAS Institute, Cary, NC).

To test the hypothesis that potential differences in growth and condition of trout were related to a distinct origin of trout food sources (sympatry and allopatry), we performed both an individual and population level analyses of trout diets. At the individual level, we described similarities in the diets using a multivariate Bray–Curtis nonmetric classification technique (Marshall and Elliot 1997; Clarke, 1993). We used PRIMER v6.1.5 software (Clarke & Gorley, 2006) to produce a similarity resemblance matrix using the Bray–Curtis similarity coefficient (Clarke & Warwick, 2001). We tested differences in trout diets between streams in sympatry and allopatry with beaver using a one‐way analysis of similarities test ANOSIM R statistic (Clarke, 1993) with 99,999 permutations. We determined which trout diet categories made the greatest contributions to dissimilarities using a similarity percentage SIMPER analysis (Clarke & Warwick, 2001). At the population level, we measured the diet niche breadth using Levin's standardized index, B_a_ (Hulbert, 1978; Krebs, 1989) and we estimated the dominance of prey categories using the index of preponderance, IP (Marshall and Elliot 1997). In addition, we compared the isotopic ratios of trout (δ^34^S, δ^15^N, δ^13^C) between streams in sympatry and allopatry with beaver using the Student's *t* test.

We tested the hypothesis that potential facilitation should be related to differences in trout food resource availability between streams in sympatry and allopatry using a Mann–Whitney *U* test of differences in the densities of macroinvertebrates. We used a nonparametric test owing to the data not meeting assumptions of normality and homogeneity of variances. In addition, we described similarities in the composition of macroinvertebrates in sympatry and allopatry with beaver using a multivariate Bray–Curtis nonmetric classification technique similar to the individual level diet analysis described above.

## 3 RESULTS

**Figure 2:**
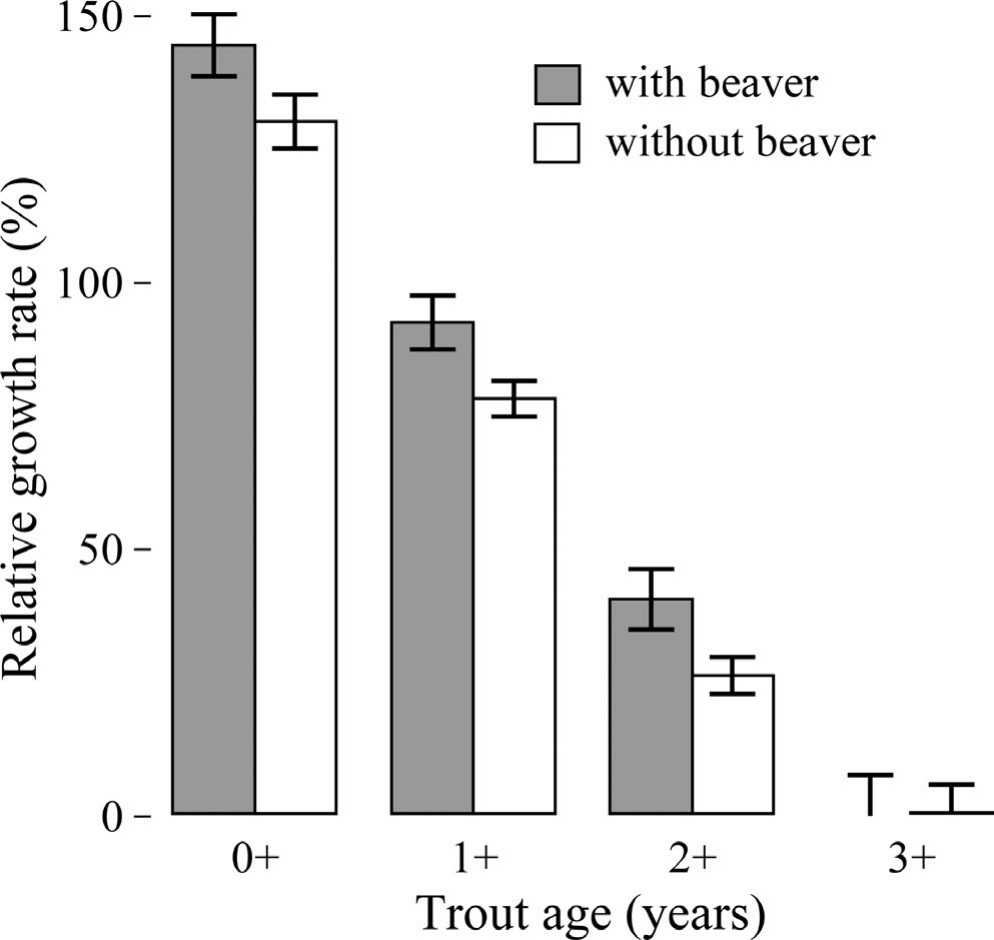
Mean estimate (±SE) of relative growth rate of Brown Trout by age with (gray bars) and without (white bars) American beaver from the general linear mixed model

Our findings show evidence for higher growth in trout populations occurring in sympatry with beavers (*F*_1,5_ = 5.62, *p* = .060; Figure 2). The average growth rate of trout was estimated to be 14% higher in streams in sympatry than in allopatry (95% CI: −1 to 30). The age of trout also played a role in affecting their growth rates, but it could represent a confounding factor on the previous marginal result (*F*_1,198_ = 372.65, *p* < .001). In sympatry, the average growth rate of age 0^+^ trout was estimated to be 145% (95% CI: 130 to 159), while the average growth rate of age 2^+^ trout was estimated to be 41% (95% CI: 26 to 55). The presence of active beaver dams did not influence trout condition indices *W*_r_ (*F*_1,5_ = 1.46, *p* = .280) or *K* (*F*_1,5_ = 1.44, *p* = .280). Similarly, trout age did not influence trout condition indices *W*_r_ (*F*_1,230_ = 0.80, *p* = .370) or *K* (*F*_1,230_ = 0.26, *p* = .610).

**Figure 3:**
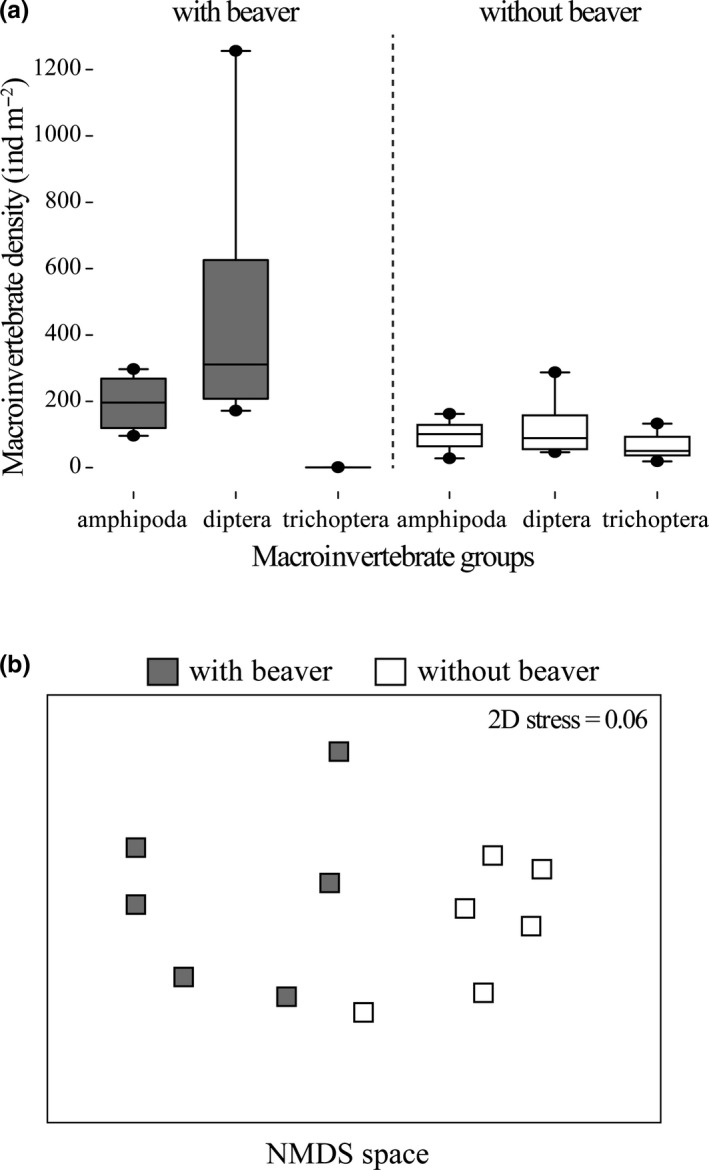
(a) Box plots of densities of most relevant macroinvertebrate groups in streams with Brown Trout in sympatry and allopatry with American beaver. The dotted line separates macroinvertebrate groups at sites with and without American beaver. (b) Nonmetric multi‐dimensional scaling (nMDS) ordination plot using Bray–Curtis similarity resemblance matrix of macroinvertebrate assemblages in streams with Brown Trout in sympatry (gray) and allopatry (white) with American beaver

Macroinvertebrate densities were higher in streams with beaver (*U* test Z = 2.49, *p = *.016; Figure 3a). In particular, diptera followed by amphipoda was the taxa with the highest densities in streams with beaver. Trichoptera was >30 times more abundant in allopatry than in sympatry, but amphipoda and diptera were around two and three times greater in sympatry than in allopatry. The composition of macroinvertebrates in sympatry was also dissimilar compared to in allopatry (*R* = 0.77, *p* < .002; Figure 3b) with trichoptera (31%), diptera (18%), and amphipoda (17%) as the main contributors to the total dissimilarity (39%). In sympatry, diptera (mean SIMPER 50%) and amphipoda (mean SIMPER 17%) had the greatest contribution to the similarity of macroinvertebrates (mean SIMPER 74%), whereas in allopatry, diptera (mean of SIMPER 27%), amphipoda (mean of SIMPER 25%), and trichoptera (mean of SIMPER 19%) were the taxa with the greatest contribution (mean SIMPER 83%).

**Figure 4:**
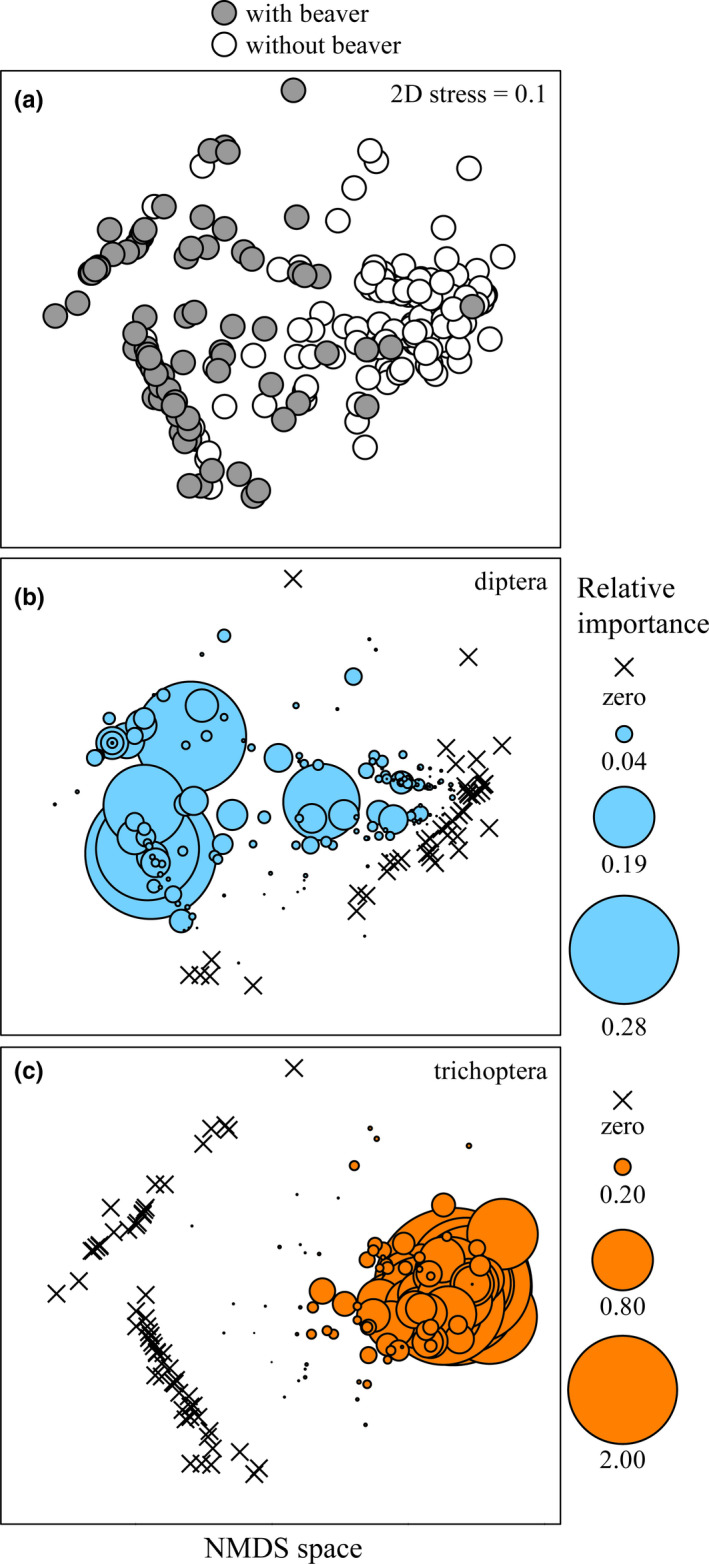
(a) Nonmetric multi‐dimensional scaling (nMDS) ordination plot using Bray–Curtis similarity resemblance matrix of Brown Trout diet in sympatry (gray circles) and allopatry (white circles) with American beaver including all stomach analyzed. (b, c) Bubble graphs from the nMDS ordination plot. The size of bubbles represents the percentage by weight %*W* for (b) diptera and (c) trichoptera in Brown Trout diets. The x‐symbol indicates absence of the respective food item. For (a, b, c), each point/circle on the plot represents an individual trout

There was a high similarity of individual diets for trout amongstreams in allopatry (*R* = 0.01, *p = .70*) and among streams in sympatry (*R* = 0.04, *p = .520*). Consequently, we pooled trout diets into two categories: allopatry and sympatry (Figure 4). Diets of trout in sympatry with beaver were dissimilar compared to in allopatry (*R* = 0.63, *p* < .001; Figure 4a) with trichoptera (37%), diptera (29%), and amphipoda (17%) as the main contributors to the total dissimilarity (74%). In sympatry (Figure 4b), diptera (mean SIMPER 37%) and amphipoda (mean SIMPER 8%) had the greatest contribution to the similarity of diets (mean SIMPER 49%), whereas in allopatry (Figure 4c), trichoptera (mean of SIMPER 54%) was the prey with the greatest contribution (mean SIMPER 63%). There were only few empty stomachs of trout in allopatry (2%) or sympatry (2%) with beaver.

**Figure 5:**
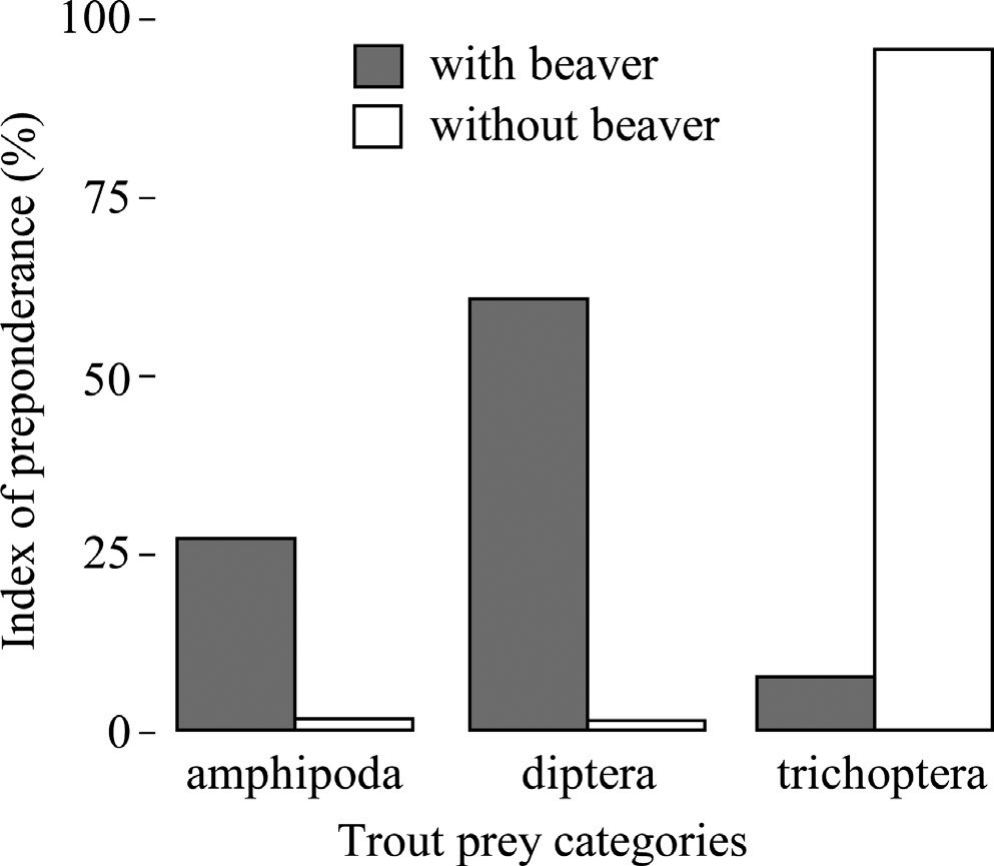
Index of preponderance (*IP*) of prey categories for all Brown Trout (>1%) with (gray bars) and without (white bars) American beaver

Similar to the individual level, the trout diet analysis at the population level showed differences between sympatry and allopatry with a wider niche breadth for trout in sympatry (B_a_ = 0.23) versus in allopatry (B_a_ = 0.02). In addition, the dominant prey types were diptera and amphipoda in sympatry, whereas in allopatry dominant prey were trichopteran (Figure 5). The calorific content of trout stomachs reflected the same results as the dominant prey types with 38% of estimated calories from amphipoda and diptera in sympatry and 73% of estimated calories from trichopteran in allopatry.

**Figure 6:**
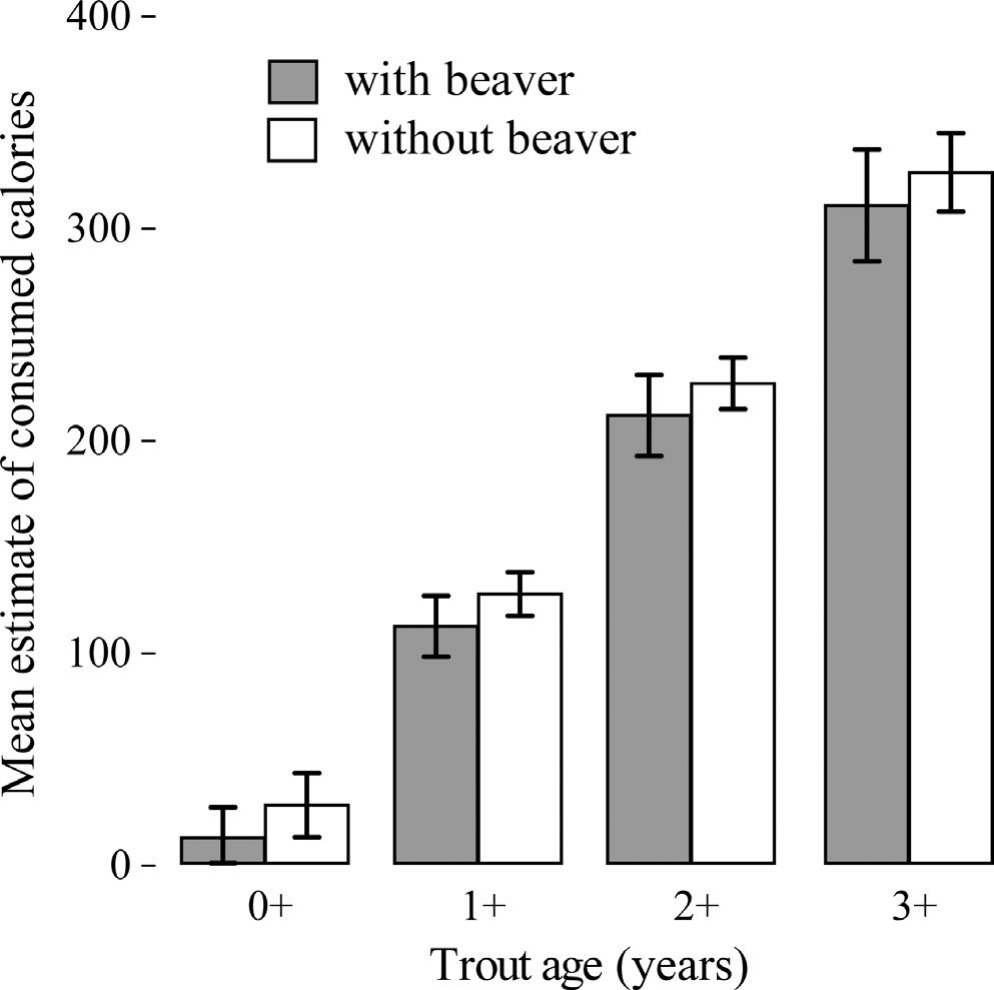
Bar chart of the mean estimate (±SE) of consumed calories by Brown Trout in sites with (gray bars) and without (white bars) American beaver by age from the general mixed model

**Figure 7:**
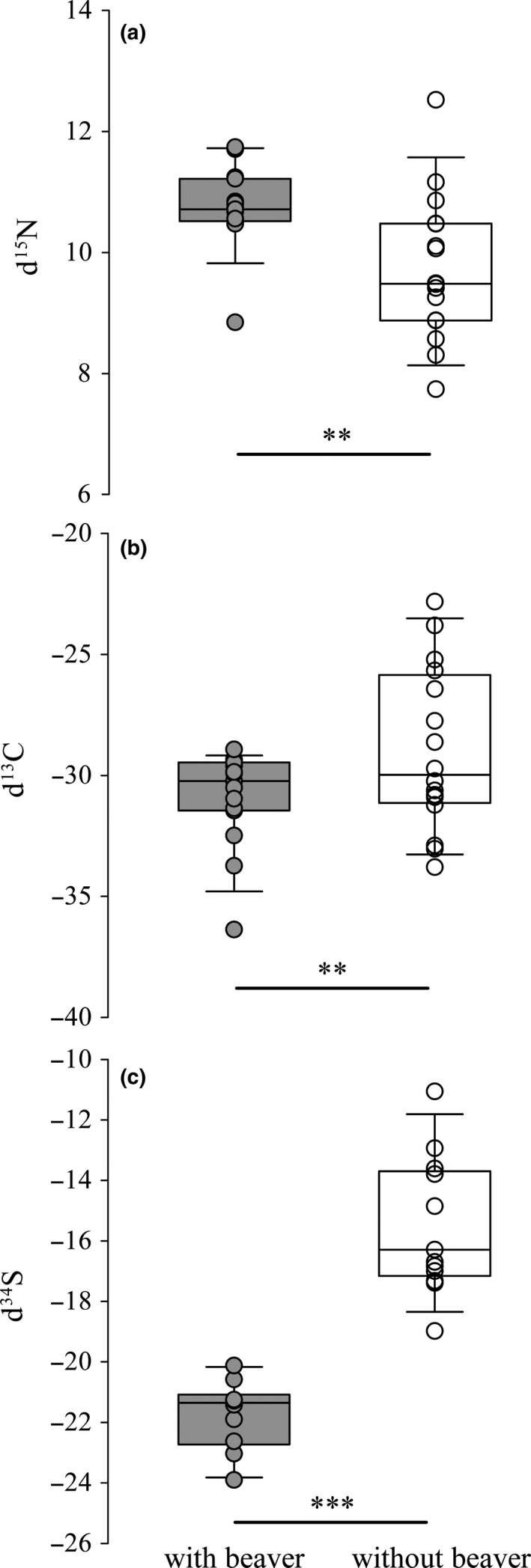
Box plots of stable isotopes of (a) nitrogen (*n* = 31), (b) carbon (*n* = 31) and (c) sulfur (*n* = 23) from Brown Trout muscle sampled from sites with (gray) and without (white) American beaver

The energetic content of consumed food was similar in sympatry compared to in allopatry with beaver (*F*_1,5_ = 0.69, *p = *.440; Figure 6). However, the age of trout played a role in affecting the energetic content of consumed food (*F*_1,274_ = 123.48, *p < *.001). For example, the energetic content of consumed food of age 1 + trout was estimated to be 113 cal (95% CI: 76 to 150) in sympatry and 128 cal (95% CI: 101–154) in allopatry whereas the energetic content of consumed food of age 3 + trout was estimated to be 311 cal (95% CI: 244–379) in sympatry and 327 cal (95% CI: 279–374) in allopatry. Further, stable isotope ratios (Figure 7) revealed that trout were more depleted in carbon (*t*_0.05,29_ = 2.904, *p* < .050) and sulfur (*t*_0.05,21_ = −7.93, *p* < .001), but more enriched in nitrogen (*t*_0.05,29_ = 2.904, *p* < .050) with beavers.

## 4 DISCUSSION

Our findings suggest that under natural whole ecosystem conditions introduced beaver exert indirect influences on the performance of non‐native trout leading to a one‐way positive interaction. Specifically, this indirect one‐way facilitation from beaver toward trout results in a marginally positive influence on trout growth. Where beaver and trout naturally coexist, there are positive effects of beavers on trout (Alexander, 1998; Hägglund & Sjöberg, 1999; Leidholt‐Bruner, Hibbs, & McComb, 1992; Sigourney et al., 2006) through increased prey availability.

Collectively, higher trout growth rates coupled with the positive association between prey and diet composition suggest that the higher macroinvertebrate density in beaver sympatry increases trout food resources. Beaver dams create profound changes in streams enhancing the input and retention of organic matter, nutrients, and other elements (Naiman & Melillo, 1984; Naiman, Melillo, & Hobbie, 1986; Pollock et al., 1995). The greater availability of food resources and suitable habitat for trout in sympatry is most likely related to the physical modifications caused by beaver. The higher macroinvertebrate production in sympatry is supported by evidence from sites where beaver are native (McDowell & Naiman, 1986; Rolauffs et al., 2001) and introduced (Anderson & Rosemond, 2007, 2010; Vila et al., 1999). Higher macroinvertebrate production provides a key food source that is used by introduced trout and native fishes (Moorman et al., 2009), and it may influence their observed higher growth rates, as is also seen in North America (Sigourney et al., 2006). In addition, trout inhabiting the impoundments created by beaver have access to refuge areas during extreme conditions in winter (Alexander, 1998). These areas usually have large woody debris that provides velocity shelter, visual isolation from competitors, and refuge from predators (Leidholt–Bruner et al. 1992; Hägglund & Sjöberg, 1999).

Our findings from stable isotope ratios suggest that the energy pathways for trout may originate from distinct food sources at sites with and without beaver (see also Peterson, Howarth, & Garritt, 1985 and McCutchan et al., 2003). The carbon pathway for trout at the base of the food web in streams without beaver seems to be linked to terrestrial inputs in comparison to streams with beaver where carbon is more depleted. The hydrological modifications made by beaver include a fundamental change in stream habitats (Figure 1) by introducing massive amounts of forest litter into streams and producing a lentic habitat where silt and anoxic areas promote microbial activity upon anoxic sediments. Likely food webs affected by beaver are anchored by decomposers with macroinvertebrates feeding primarily on bacterial/fungal biofilms, whereas in absence of beaver food webs rely on macroinvertebrates feeding primarily on primary producers based on algal biofilms. Further, trout sulfur isotopic ratios are more depleted in sympatry with beaver than in allopatry, reflecting a different and longer pathway from microbial cycling in anoxic sediments typically described for beaver impoundments in native and introduced systems (Anderson et al., 2020; Naiman & Melillo, 1984; Naiman et al., 1986). In general, our sulfur values are extremely depleted compared with other ecosystems (McCutchan et al., 2003) likely due to naturally short food webs in sub‐Antarctic freshwater systems. For example, although few native fishes are present in these sub‐Antarctic systems, introduced Brown Trout are the only fish at our study sites (Vila et al., 1999). These freshwater food webs often stop at the invertebrate herbivore level, and the decomposer cycle assumes a dominant role in the biological cycling of nutrients (Anderson et al., 2020; Smith, 1985).

Differences in growth rates of trout in sympatry and allopatry with beaver may be influenced by other factors besides food availability and diet composition. Among them are density‐dependent factors (Sundström, Kaspersson, Näslund, & Johnsson, 2013), potential biases in back‐calculated growth rates (Francis, 1990), and differences in thermal regimes between stream sections with and without beaver (Majerova, Neilson, & Roper, 2020). Unfortunately, we were not able to evaluate the importance of these factors in our study sites owing to logistical reasons, including (a) difficulties obtaining population estimates in large, deep beaver ponds, and (b) back‐calculated rates of growth could be biased if the entire length range of the population is not represented. We encourage future efforts using mark–recapture methods to estimate population size and sensor instrumentation of stream networks in order to test the importance of density‐dependent factors and thermal regimes of streams on trout growth in invaded systems.

Trout in allopatry may have higher energetic costs due to higher water velocities in flowing streams than in beaver impoundments (Shuler, Nehring, & Fausch, 1994; O’Connor and Rahel 2009). Even though there are similar levels of energy consumption in trout stomachs between sites with and without beaver, growth rates are marginally lower in allopatry. It is possible that we may have underestimated the energy contents in trout stomachs, especially in sympatry because the most abundant resources (i.e., diptera and anphipoda) may have higher rates of digestion as soft‐body prey items (Hyslop, 1980). Further, we show evidence of similar body condition of trout (*W*r, *K*) in sympatry and allopatry. Condition indices have been used as an indicator of recent feeding activity, body energy reserves, and relative abundances (Arismendi, Penaluna, & Soto, 2011; Blackwell, Brown, & Willis, 2000; Froese, 2006). The reduced diet breadth in allopatry can be explained in two ways: trout may be food limited and forced to feed on a restricted diet, or conversely they are feeding optimally, and it is only under sympatry where they are forced to include other (nonoptimal) items in their diet.

In summary, beaver are ecosystem engineers that play a fundamental role in positively influencing the growth of invasive trout as seen here, but they also enhance mammal (Crego et al., 2016) and riparian plant (Anderson, Griffith, Rosemond, Rozzi, & Dollenz, 2006) introductions and provide refugia for native fishes in other systems (Moorman et al., 2009). Consequently, a special importance should be focused on them because of their capacity to affect the structure and functioning of complete ecosystems by influencing water and nutrient dynamics (Ulloa et al., 2012), trophic interactions (Anderson & Rosemond, 2010; Anderson et al., 2020), and disturbance regimes (Chapin et al., 1997; Pollock et al., 1995). We provide evidence that two species with a particular ecosystem function in their native range may promote similar interactions in invaded systems even though they have not naturally occurred in sympatry. Hence, consideration of ecological and evolutionary histories among introduced species may improve the forecasting of invasion success of new species introductions and guide the identification of vulnerable ecosystems.

## CONFLICT OF INTEREST

The authors declare no conflict of interest associated with this manuscript.

## AUTHOR CONTRIBUTION

**Ivan Arismendi:** Conceptualization (lead); Data curation (lead); Formal analysis (lead); Funding acquisition (lead); Investigation (lead); Methodology (lead); Project administration (lead); Resources (lead); Supervision (equal); Visualization (lead); Writing‐original draft (lead); Writing‐review & editing (lead). **Brooke E Penaluna:** Formal analysis (equal); Investigation (equal); Writing‐review & editing (supporting). **Carlos G Jara:** Conceptualization (supporting); Data curation (supporting); Investigation (supporting); Methodology (supporting); Resources (supporting); Supervision (supporting); Writing‐original draft (supporting); Writing‐review & editing (supporting).

## Supporting information

Appendix S1

## Data Availability

The data that support the findings of this study have been deposited in Dryad https://doi.org/10.5061/dryad.7wm37pvqr
