# Supplementary material for: Introduced beaver improve growth of non‐native trout in Tierra del Fuego, South America
Source: Ecol Evol. 2020 Aug 17;10(17):9454–65. doi: 10.1002/ece3.6636 (PMC7487219; doi:10.1002/ece3.6636)
Supplement: Supplementary file 1 — Appendix S1 [file ECE3-10-9454-s001.docx]

**Appendix 1.** Mean, min and max of monthly stream temperature (°C) and stream flow (m^3^ s^-1^) at Rio Grande gauge station. Source: Dirección General de Aguas, Chile <https://snia.mop.gob.cl/BNAConsultas/reportes>,
